# Tumor-Associated Neutrophils and Macrophages—Heterogenous but Not Chaotic

**DOI:** 10.3389/fimmu.2020.553967

**Published:** 2020-12-02

**Authors:** Ling Wu, Xiang H.-F. Zhang

**Affiliations:** ^1^ Lester and Sue Smith Breast Center, Baylor College of Medicine, Houston, TX, United States; ^2^ Dan L. Duncan Cancer Center, Baylor College of Medicine, Houston, TX, United States; ^3^ Department of Molecular and Cellular Biology, Baylor College of Medicine, Houston, TX, United States; ^4^ McNair Medical Institute, Baylor College of Medicine, Houston, TX, United States

**Keywords:** tumor-associated neutrophils, tumor-associated macrophages, metastasis, tumor microenvironment, tumor evolution

## Abstract

Tumor-associated macrophages (TAMs) and tumor-associated neutrophils (TANs) have been extensively studied. Their pleotropic roles were observed in multiple steps of tumor progression and metastasis, and sometimes appeared to be inconsistent across different studies. In this review, we collectively discussed many lines of evidence supporting the mutual influence between cancer cells and TAMs/TANs. We focused on how direct interactions among these cells dictate co-evolution involving not only clonal competition of cancer cells, but also landscape shift of the entire tumor microenvironment (TME). This co-evolution may take distinct paths and contribute to the heterogeneity of cancer cells and immune cells across different tumors. A more in-depth understanding of the cancer-TAM/TAN co-evolution will shed light on the development of TME that mediates metastasis and therapeutic resistance.

## Introduction

Tumors are heterogeneous at multiple levels. Genomic and transcriptomic profiles classifies many cancers into different intrinsic subtypes (1–5). Individual tumors consist of not only neoplastic cells but also a variety of stromal cells and extracellular matrix components that together constitute tumor microenvironment (TME) that determines tumorigenesis and tumor progression. Even for cancer cells within the same tumor, yet another layer of heterogeneity exists among different cells due to clonal evolution or variable status of differentiation. These different levels of heterogeneity represent a major obstacle against effective therapies that can be applied to most patients.

Neutrophils and macrophages are the most abundant immune cells that infiltrate tumors (6–8). The crosstalk between tumor cells and the infiltrated neutrophils and macrophages can contribute to drive tumor growth and metastasis. Recent research suggests that frequencies of tumor-associated macrophages (TAMs) and tumor-associated neutrophils (TANs) can vary across different breast cancers, thereby forming a previously unappreciated level of heterogeneity across patients, but extrinsic to cancer cells. This heterogeneity appears to be somewhat inheritable but may become altered when tumors are subjected to therapeutic interventions. It is compelling to hypothesize that cancer cells co-evolve with TAMs and TANs: whereas TAMs or TANs confer selective advantages to cancer cells with specific properties, different cancer cell clones also preferentially recruit certain myeloid cell populations, thereby forming a loose symbiosis-like relation that is highly context-specific.

Both TAMs and TANs have immunosuppressive functions and are known to modulate anti-tumor immunity, which are covered by outstanding reviews in this issue or elsewhere (7–10). In this review, we focus on evidence showing that TAMs and TANs directly participate in tumor initiation, proliferation, and metastasis. We will highlight the heterogeneity of breast cancers and how this heterogeneity can reciprocally shape the surrounding tumor microenvironments. Finally, we will discuss our lack of knowledge in direct cancer-myeloid interactions that are selective based on different cancer-intrinsic properties and myeloid subpopulations. Although the principle and hypothesis may not be cancer type–specific, we will use breast cancer as a representative in the final discussion to integrate our knowledge and exemplify future directions.

## Tumor-Associated Neutrophils

Neutrophils are the first line of defense of our immune system, abundantly circulating in peripheral blood. When foreign pathogens invade human bodies, neutrophils are quickly recruited to the site of inflammation to exert antimicrobial moieties (11, 12). Neutrophils make up a considerable proportion of the immune cells infiltrated in primary tumors including lung, breast, gastric and others and are associated with poor overall survival and recurrence-free survival (6, 13). Meta-analysis has shown that a high density of intratumoral neutrophils are independently associated with unfavorable survival, whereas the peritumoral and stromal neutrophils were not (14). Traditionally believed as short-lived, neutrophils have been shown to have longer lifespans in tumor bearing settings, likely due to support from tumor secreted cytokines (15, 16). Tumor associated neutrophils (TANs) actively participate in various steps of tumor progression and have been reported to have both antitumor and pro-tumor roles. Direct cytotoxicity of TANs has been found to inhibit tumor progression and metastasis (17–19), however a larger number of pro-tumor functions have been uncovered. These include angiogenic switch, promotion of migration and invasion, as well as exertion of immunosuppression (7). Like TAMs, TANs of different roles on tumors were classified as N1 (antitumor) or N2 (protumor). A study by Fridlender et al. showed that blockade of TGF-beta increased recruitment of anti-tumor pro-inflammatory neutrophils. These neutrophils exhibited nuclei that were hypersegmented compared to neutrophils present under TGF-β suffice conditions. These two different kinds of neutrophils are termed as TAN N1 or N2 (20). It remains to be elucidated whether the N1 and N2 statuses result from polarization or different degrees of maturation. Regardless, this and related studies demonstrated that neutrophils are not a homogenous entity and should be studied in a context dependent manner. In the following paragraphs, we will focus on the roles of TANs in specific aspects of tumor progression (**Table 1**).

**Table 1:** The role of tumor-associated neutrophils in cancer.

Function	Identification Markers	Tumor model	Experimental system	Reference
Tumor initiation	CD11b^+^ Ly6G^+^; MPO+	*Apc* ^Min/+^, *Ah*−*CreER; Apc* ^F/+^ *; Pten* ^F/F^, *K14−CreER;Kras* ^G12D/+^ spontaneous models, carcinogen induced model	Mouse, *in vivo*	(21)
	CD11b^+^ Ly6G^+^	Diethynitrosamine induced hepatocellular carcinoma, nfkb1^−/−^	Mouse, *in vivo*	(22)
	Mpx	Gfap-Kras^G12V^ astrocytes	Zebra fish larvae	(23)
Tumor proliferation	NE	TE-1, TE-7, TE-8, TE-12, TE-13	Human, *in vitro*	(24)
	NE	*loxP*-Stop-*loxP* K-ras^G12D^	Mouse, *in vivo*	(25)
	CD11b^+^ Ly6G^+^ Ly6C^+^	PC3	Human, *in vivo*; human, *in vitro*	(26)
	Cytoplasic granule morphology MMP9^+^	MMP-9^−/−^ HPV16 model	Mouse, *in vivo*	(27)
Proliferation Metastasis	MPO	D2.0R	Mouse, *in vivo*	(28)
Angiogenesis	Chick heterophils, gradient centrifugation	Chick embryos with 3D collagen grafts	Chick, *in vivo*; Human, *in vivo*	(29)
	Ly6G^+^	Chick embryos with 3D collagen grafts; PC3, L929, B16, LLC	Chick, *in vivo*; Mouse, *in vivo*, Human, *in vivo*	(30)
	CD11b^+^ Gr1^+^	EL4, LLC, B16F1, T1B6	Mouse *in vivo*	(31)
	CD11b^+^ Gr1^+^	RIP-Tag2 model; HM7	Mouse, *in vivo*; human, *in vivo*	(32, 33)
Invasion and migration	Gradient centrifugation	AsPC-1, HepG2, MDA-MB-468	Human, *in vitro*	(34–36)
EMT	Gradient centrifugation, NASDCL, elastase	PDAC biopsies, T3M4, HuH7	Human, patient samples; Human, *in vitro*	(37)
	CD66b^+^	MKN45, MKN74	Human, *in vitro*	(38)
	CD66b^+^	Lung carcinoma samples	Human, patient samples	(39)
	CD11b^+^CD66b^+^	MCF-7	Human, *in vitro*	(40)
CTC proliferation	Ly6G^+^, Wright-Giemsa staining	BR16-GFP	Human, *in vivo*	(41)
Extravasation	CD11b^+^	C8161.CI9, 1205Lu; A375, MDA-MB-231	Human, *in vitro*	(42, 43),
Metastasis	Ly6G^+^	4T1	Mouse, *in vivo*	(44)

## The Impact of TANs in Human Cancers

The correlation between neutrophils and cancer prognosis remains to be precisely characterized (**Table 2**). In peripheral blood, high neutrophil to lymphocyte ratio (NLR) is associated with worse prognosis in patients with a variety of cancers, including breast cancer (13, 45). In many studies, the infiltration of TANs in cancer follows a similar trend and is associated with poor clinical outcomes (6, 13). In terms of microenvironmental characteristics, TANs were found to be inversely correlated with T cell infiltration and positively correlated with angiogenesis, consistent with pro-tumor roles (57, 58). However, there were also studies showing TANs as good prognostic factors in colorectal cancer, squamous cell carcinoma and invasive ductal breast carcinoma. The antitumor effects of neutrophils may be mediated through direct killing or coordinating with adaptive lymphocytes. These seemingly controversial results, sometime even within the same cancer type, might be derived based on different markers used. For example, frequency of high CD66^+^ neutrophil is positive correlated with CRC malignancy, while myeloperoxidase (MPO^+^) neutrophils exhibited the opposite trend as good prognosis factor (51, 52). These discrepancies highlight the urgent need for precise characterization of the heterogeneous “neutrophil” populations. The current marker system is clearly insufficient. The functionally distinct subpopulations need to be identified and separated, in both experimental and clinical studies.

**Table 2:** The clinical relevance of TANs in human cancers.

Type of Cancer	Marker	Correlation	Reference
Breast cancer	Peripheral blood neutrophil to lymphocyte ratio (NLR)	Short- or long-term mortality	(45)
Renal cell carcinoma	CD66b^+^	Short RFS	(46)
Melanoma	CD66b^+^ and CD123^+^ DCs	Poor prognosis	(47)
Head and neck cancer	Polymorphonuclear granulocytes	Poor survival	(48)
Hepatocellular carcinoma	CD66b^+^	Early recurrence and decreased PFS/OS	(49)
Colorectal cancer	CD66b^+^	Better prognosis	(50)
Colorectal cancer	CD66b^+^	Poor prognosis	(51)
Colorectal cancer	MPO^+^	Better prognosis	(52)
Gastric adenocarcinoma	CD15^+^	Independent and unfavorable factor in prognosis	(53)
Human gliomas	CD15^+^ and MPO^+^	High tumor grade	(54)
Pancreatic adenocarcinoma	Polymorphonuclear granulocytes	More malignant subtype	(55)
Pancreatic adenocarcinoma	CD66b^+^	Associated with shorter survival along with pan-macrophages and M2 macrophages	(56)

## TANs in Tumor Initiation

Inflammation-induced damage promotes tumorigenesis independent of cancer-intrinsic genetic mutations. Studies showed that neutrophils are more frequently recruited to tumor-prone tissue through chemotaxis (21, 59). Using three genetically engineered spontaneous tumor mouse models to mimic the tumorigenesis in human, Jamieson et al. found that CXCR2 ligands were upregulated in all three models, including intestinal adenoma (*Apc* ^Min/+^), the invasive intestinal adenocarcinoma (*Ah*−*CreER; Apc* ^F/+^ *; Pten* ^F/F^) and the spontaneous oral papilloma (*K14−CreER;Kras* ^G12D/+^) model. CXCR2 inhibition or deficiency suppressed tumor formation in *Apc* ^Min/+^ model and *Ah*−*CreER; Apc* ^F/+^ *; Pten* ^F/F^ model, respectively. Administration of carcinogens failed to induce papilloma or adenoma in CXCR2 deficient mice, in which neutrophils trafficking was significantly impaired. Depletion of Ly6G^+^ cells using anti-Ly6G antibodies showed a similar inhibitory effect of tumorigenesis in both chemical-induced and spontaneous models. Although the detailed mechanism was not discussed, myeperoxidase (MPO) was detected on neutrophils, which might link the reactive oxidative stress induced by neutrophils to tumorigenesis (21). The genotoxic substances released by neutrophils can initiate a carcinogenic response by inflicting DNA damage on epithelial cells. Neutrophils was reported to stimulate ROS and telomere DNA damage in hepatocytes. Upon depletion of hepatic neutrophils by anti-Ly6G (1A8) antibody, diethynitrosamine (DEN) induced hepatocellular carcinoma was attenuated. Anti-oxidant treatment led to protection against progression of DEN induced hepatocellular carcinoma (22). Another study reported that neutrophils were recruited to Kras^G12V^-expressing astrocytes in an optical transparent larva zebrafish model of glioblastoma by CXCR1. The proliferation of these tumor-initiating astrocytes was also blunted when neutrophil chemotaxis signaling CXCR1/2 was inhibited (23). Thus, it appears that TANs can enhance tumor initiation either through exerting genotoxicity by inducing ROS or potentiating the tumor initiating cells.

## TANs in Tumor Proliferation

Several molecules (NE, MMP9, Bv8) expressed on neutrophils can mediate their positive roles in tumor proliferation. As a serine protease, the proteolysis ability of neutrophil elastase (NE) is able to release growth factors from cancer cells. In an *in vitro* esophageal cell line model, NE treatment led to a rapid release of TGF-α, PDGF and VEGF along with EGFR phosphorylation. Increased cell proliferation and invasion was also observed in all five cell lines tested (24). In a *loxP*-Stop-*loxP* K-ras^G12D^ (LSL-K-ras) model of mouse lung adenocarcinoma, Houghton et al. showed that neutrophil elastase is endocytosed by tumor cells where it degrades IRS-1 and skews the PI3K toward tumor proliferation (25). Hammes et al. demonstrated NE is produced by infiltrating immune cells using live imaging of nude mice bearing PC3 tumors. Inhibition of NE could suppress PC3 xenograft growth. Mechanistically, NE activates MAPK and its downstream signaling in PC3 cells (26). Inhibition of NE by Elafin also shows tumor suppressing activity by inducing Retinoblastoma pathway dependent cell cycle arrest and elevated apoptotic cell death (60). Coussens et al. showed MMP9 was mainly expressed by neutrophils, macrophages and mast cells. MMP9 knockout mice exhibited reduced keratinocyte hyperproliferation and bone marrow transplantation of MMP9 expressing cells can restore the tumor growth in these mice (61). The NE and MMP9 loaded on the neutrophil extracellular traps (NETs) were also found to awaken dormant cancer cells to proliferate through sequential cleavage of laminin in the extracellular matrix of the dormant cancer cells to activate integrin and YAP signaling (28). Taken together, the protease-enriched secretome of TANs appear to be able to activate several growth factor pathways at different levels to enhance proliferation.

## TANs in Angiogenesis

MMP9 produced by neutrophils in the tumor microenvironment was also found to be strongly associated with the tumor angiogenesis. Using a quantitative non-tumor *in vivo* model to induce angiogenesis in 3D collagen rafts, Quigley et al. revealed that the angiogenesis is facilitated by the MMPs of the infiltrated inflammatory cells including heterophils. And the potent angiogenic characteristic was related to the active form of MMP-9 that was free of tissue inhibitor of metalloproteinases (TIMP) (29). They also showed in a later study that tumor infiltrated neutrophils are a major source of MMP-9 and is highly linked to tumor angiogenesis in a PC3 orthotopic prostate cancer xenografts in NOD/SCID mice (30). Using a RIP-Tag2 model of pancreatic islet carcinoma, Bergers et al. revealed the specific angiogenesis role of MMP-9 by releasing VEGF from normal and hyperplastic pancreatic islets (62). The absence of MMP-9 function reduced the angiogenic switching and the growth of tumor cells. An increased intratumor infiltration of neutrophils was correlated with glioma grade as well as the resistance to anti-VEGF therapy (31, 63). Structurally similar to VEGF, G-CSF induced Bv8 secretion by bone-marrow-derived cells was implicated in tumor angiogenesis by neutrophils. Shojaei et al. elucidated the role of Bv8 in RIP-Tag angiogenic switching, where systemic depletion of Bv8 by anti-Bv8 antibody at early stage significantly reduced angiogenic islets number as well as the homing of CD11b^+^ Gr1^+^ cells to the emerging neoplastic lesions (32, 33). Anti-CSF or anti-Bv8 confers additional effect on anti-VEGF therapy. Therefore, both MMP-9 and Bv8 are responsible for the angiogenic effects of TANs.

## TANs in Metastasis

The roles of TANs in metastasis are pleiotropic and highly context dependent. The 13762NF rat mammary adenocarcinoma clones with varying metastatic potentials showed a dose-dependent increase of invasion in a reconstituted basal membrane invasion culture system when co-cultured with neutrophils (a.k.a., polymorphnuclear leukocytes or PMN) from tumor bearing rats. These clones also exhibited increased lung metastases *in vivo* when co-injected with the tumor elicited neutrophils compared with those PMNs from normal rats (64). The same group also discovered later that bone marrow cellularity and myeloid erythroid ratios positively correlated with the metastatic potentials of the tumors these rats bared (65). Jung et al. showed an increased neutrophil extracellular traps formation in blood sample after co-cultured with AsPC-1 cells. Using *in vitro* Boyden chamber model, NETs increased migration and invasion of AsPC-1 cells than intact neutrophils alone, which can be inhibited by histone binding agents, some DNA-degrading enzyme as well as Toll-like receptor neutralizing antibodies (34). The interactions between cancer cells and neutrophils are not unidirectional. Reciprocally, the survival of neutrophils can be enhanced by tumor supernatant from hepatocellular, cervical, colorectal and gastric carcinoma cell lines. This effect can be mimicked by Hyaluronan fragments. Blocking the interactions between HA and TLR4 on neutrophils could mitigate this pro-survival of neutrophils as well as the migration of cancer cells (35). Strell et al. found that MDA-MB-468 cells that secreted IL-8 and GRO-α increased the migratory activity of neutrophils and recruitment to tumor cells to enable cell-cell interaction, which led to the binding of β_2_-integrins expressed by neutrophils and its receptor ICAM-1 on MDA-MB-468 cells. The focal adhesion molecules including FAK were then phosphorylated by SRC kinase and the p38 MAPK was activated by Rho kinase. Eventually, the migration of tumor cells was increased (36). These studies highlighted the importance of the crosstalk between neutrophils and cancer cells during tumor progression, and demonstrated effects of neutrophils on pathways related to migration and invasion.

TANs can trigger epithelial-mesenchymal transition (EMT). Neutrophil elastase cleavage of E-Cadherin induced EMT in pancreatic and liver cancer cell line *in vitro*. Co-culture with either neutrophils or NE could induce rapid cell dyshesion and E-Cadherin degradation as early as 3 h after co-culture. In parallel, the upregulation of TWIST, translocation of β-catenin into the nucleus, nuclear expression of ZEB1, and the downregulation of keratin was also observed. Using PDAC biopsy samples, Steffen et al. showed the positive correlation of PMN infiltration with the EMT status using ZEB1 or nuclear β-catenin expression (37). Li et al. found that neutrophils were enriched in gastric cancer tissues in patients, especially in the tumor invasive edge. Coculturing of tumor associated neutrophils with gastric cells *in vitro* significantly decreased E-cadherin expression along with the upregulation of vimentin and ZEB1. The migration and invasion of the gastric cancer cells were also increased. This effect was related to the IL-17a secreted by neutrophils. Blocking IL-17a with neutralizing antibody inhibited the TAN-stimulated activities in gastric cancer cells (38). Hu et al. showed a negative association of intratumoral CD66^+^ PMNs expression with the E-cadherin expression. Neutrophils induced EMT was observed *in vitro* accompanied by enhanced migration of tumor cells, where TGF-β/Smad signaling was initiated and in part related to this process (39). A study by Wang et al. demonstrated that it was the neutrophils isolated from breast tumors but not from peripheral blood can significantly promote migration and invasion of a panel of breast cancer cell lines *in vitro*. MCF7 cells cultured with 30% conditioned medium from tumor infiltrating neutrophils showed mesenchymal morphology along with the downregulation of E-cadherin as well as the upregulation of Twist expression. These effects were abrogated by blocking TIMP-1 of neutrophils. Reciprocally, MCF7 cells that underwent EMT could stimulate the neutrophil expression of TIMP-1 through CD90 in a contact dependent manner (40).

Neutrophil derived enzymes also promote tumor intravasation besides angiogenesis. Using a chick embryo spontaneous intravasation assay, Bekes et al. demonstrated an essential role of proMMP9 protease in modulating certain variants of PC3 or HT-1080 cell intravasation *in vivo* (66). The neutrophils expressing MMP9 were recruited to primary tumors of highly disseminating variants to enhance their intravasation and angiogenesis. Blocking neutrophil influx by anti-IL-8 antibodies diminished both intravasation and angiogenesis.

After intravasation, it is inevitable for circulating tumor cells to encounter leukocytes. Szczerba et al. found a rare but consistent CTC-WBC clusters in peripheral blood samples from both breast cancer patients and tumor bearing mice. Most of these clusters are CTC-neutrophil clusters, which correlates with significantly worse progression-free survival in patients. Compared to CTC alone, CTCs from clusters were observed to be more proliferative with a marked enrichment in positive regulators of cell cycle and DNA replication (41).

Extravasation is a key step for disseminated cancer cells to seed in the distant organs. Neutrophils were seen to facilitate this process. Attracted by IL-8 secreted by melanoma, neutrophils interacted with the melanoma cells through β_2_-integrin ICAM-1 and promoted docking along vascular endothelium. Blocking IL-8 secretion from these melanoma cells significantly decreased extravasation (42). Chen et al. employed an *in vitro* multiplexed microfluidic model of human microvasculature to observe in real-time the physiologically relevant transportation of circulating cells in a high spatial resolution. Co-injection of melanoma cells with LPS stimulated human PMNs resulted in the quick formation of tumor cell-PMN heterotypic aggregates along the endothelial under flow by both mechanical trapping and neutrophil-endothelial adhesions. By secreting IL-8, PMNs were chemotactically confined by tumor derived CXCL-1, which enhanced the extravasation of adjacent melanoma or breast cancer cells through a modulation of the endothelial barrier by IL-8. Using a neutralizing antibody against IL-8 could abrogate both PMN sequestering and the extravasation of tumor cells. Similarly, the inflamed PMNs exhibited confined migration and enhanced tumor cell extravasation in zebrafish embryos (43). The adhesion between neutrophils and disseminated tumor cells also plays a role when tumor cells arrived the organ of metastases. Clusters of neutrophil and H-59 Lewis lung carcinoma cells were seen in the liver sinusoid. This interaction was mediated by Mac-1 and ICAM-1 (67). Using two clones with different metastasis potentials from same tumor, Park et al. showed that 4T1 cells the clone with high metastasis potential recruited more neutrophils to primary tumor compared to 4T07 which have less metastasis potential. More neutrophil extracellular matrix was also found in lungs of mice injected with 4T1 cells through tail vein. Enzymatic digestion of NETs as well as anti-G-CSF antibody blocked migration and invasion *in vitro* using three different cancer cells. An intraperitoneal injection of DNase I-coated nanoparticles could prevent lung metastases in mice which received an intravenous injection of 4T1 cells (44). Neutrophils were also found to participate in the awakening of dormant cancer cells. A study from the same group showed that under inflamed conditions, NETs could awaken dormant D2.0R cells and increase metastases in mice. Neutrophil related proteases NE and MMP9 loaded on NETs’ DNA scaffolding can sequentially cleave the extracellular matrix protein laminin, which reveals an epitope to trigger proliferation of dormant cancer cells through integrin activation and FAK/ERK/YAP signaling. A blocking antibody against remodeled laminin could prevent or reduce inflammation induced dormant cancer cells awakening (28).

## Tumor-Associated Macrophage (TAMs)

Differentiated from mononuclear phagocyte lineage, macrophages are a tissue-resident cell type that play a vital role in regulating immune response to maintain tissue homeostasis and organ development. Macrophages are found as key components of the infiltrating leukocytes in various types of tumors, which are considered as wounds that never heal. TAMs have been reported to actively participate in almost every step of tumor progression including tumor angiogenesis, invasion, migration, colonization at secondary organs as well as immune suppression (**Table 3**). The association between their frequency and expression patterns and poor clinical outcomes has been reported in most of the studies focusing on the clinical implications of TAMs. Bingle et al. showed in a meta-analysis that increased macrophage infiltration frequency in primary tumors was associated with poor prognosis in most of the breast cancer cases (90). Studies from Beck and Campbell linked proliferating macrophages and their related signaling like colony-stimulating factor 1 with high grade, malignant subtype as well as poor clinical outcome (91, 92). However, multivariate model analysis by Mahmoud et al. showed that overall macrophage number (CD68^+^) was not an independent prognostic marker, which shed light on the heterogeneity and plasticity of TAMs (93).

**Table 3:** The role of tumor-associated macrophages in cancer.

Function	Identification Markers	Tumor model	Experimental system	Reference
Tumor initiation	F4/80^+^	Mdr2^−/−^ spontaneous model	Mouse, *in vivo*	(68)
	CD11b^+^ F4/80^+^	Stat3-IKO spontaneous model	Mouse, *in vivo*	(69)
Angiogenesis	CD68^+^	Breast carcinoma samples	Human, patient samples	(70)
	F4/80^+^	MMTV-PyMT/LysMCre+/VEGF^f/f^ spontaneous model	Mouse, *in vivo*	(71)
		Breast tumor samples	Human, patient samples	(72)
	CD11b^+^ F4/80^+^	E0771, LLC	Mouse, *in vivo*	(73)
	CD68^+^	K14-HPV16 spontaneous model	Mouse, *in vivo*	(74)
	F4/80^+^ Tie2^+^	PyMT	Mouse, *in vivo*	(75)
Migration and invasion	CD11b^+^/Gr1^mid/low^	MC38, LLC	Mouse, *in vitro*	(76)
	F4/80^+^	MMTV-PyMT	Mouse, *in vivo*	(77)
	CD68^+^ CD163^+^	THP-1, patient samples	Human, patient samples, human, *in vitro*	(78)
	CD11b^+^Gr1^-^F4/80^+^	MMTV-PyMT	Mouse, *in vivo*	(79)
	CD68^+^ CCL18^+^	MDA-MB-231	Human, *in vitro*	(80)
	CD68^+^; CD68^+^ CD163^+^, CD206^+^	SKBR3, MDA-MB-231; SW48	Human, *in vitro* Human, *in vitro*	(81, 82)
Intravasation	BAC1.2F5 macrophage cell line	MDA-MB-231	Human, *in vitro*	(83)
Intravasation	MRC1^+^/CD11b^+^/F4/80^+^/CD11c^–^	MMTV-PyMT	Mouse, *in vivo*	(84)
Extravasation, metastasis	CD11b^+^ F4/80^+^	Met-1	Mouse, *ex vivo*; Mouse, *in vivo*	(85, 86)
Metastasis	CD11b^+^ F4/80^+^	E0771-LG, Met-1,	Mouse, *in vivo*	(87)
EMT, metastasis	CD68^+^, CD206^+^, HLA-DR	MCF-7,	Human, *in vitro*; Humanized mouse model, *in vivo*	(88)
Anti-metastasis	Ly6C^+^	MT/ret^+/−^ spontaneous model	Mouse, *in vivo*	(89)

In an oversimplified model, macrophages polarize to two opposite states. M1 macrophages are known as classically activated macrophages, which are activated by Th1 cytokines like interferon-gamma, or together with bacterial components. These M1 macrophages exert anti-microbial properties by secreting cytotoxic molecules (e.g. reactive oxygen species and nitrogen intermediates) and pro-inflammatory cytokines (e.g., IL-6, IL-12, IL-23, TNF). As alternatively activated macrophages, M2 macrophages are activated by Th2 cytokines (e.g. IL-4, IL-10, and IL-13), which typically attenuate inflammation, promote wound healing, angiogenesis and tissue remodeling (94, 95). Polarization towards M1 or M2 requires the activation of ERK, NF-κB, and STAT1 signaling or STAT3 and STAT6 pathway, respectively. In fact, these two polarization states serve as the boundaries for a spectrum of activation states which reflects the complex tissue microenvironment that can induce simultaneous activation of different signaling pathways.

There are two sources for tumor associated macrophages. One source is from circulating Ly6C^+^ CCR2^+^ monocytes that enter tissues through the adherence of activated integrins (96). The other source is from tissue resident macrophages that originated from CXC3CR1^+^ Kit^+^ erythromyeloid progenitors from yolk sac or murine fetal liver independent of bone marrow (97, 98). Tumor associated macrophages tend to exhibit an M2-polarized state with impaired antigen presentation and tumoricidal capacity and high expression of angiogenic factors, tissue remodeling metalloproteases, and cathepsins. The polarization of TAM is not only regulated by intrinsic signaling, but also shaped by the complex immune and stromal cells in tumor microenvironment as well as the cancer cells. This complex interaction makes the polarization of TAM change over the dynamic evolution of microenvironment milieu. High production of inflammatory molecules from M1 macrophages may support neoplastic transformation in the early stage of tumorigenesis. However when a tumor was established, M2 macrophages can suppress immune surveillance and remodel tissue matrix to promote tumor progression (99). Besides the temporal change in the polarization status, macrophages phenotypes differs even within different areas of the same tumor. Two distinct tumor microenvironments were found in the same orthotopic mammary tumor. Perivascular TAMs showed stronger migration compared to those in avascular regions. Large number of perivascular macrophages at mouse mammary tumor margins could interact with cancer cells and migrate together (100). Macrophages within the tumor mass express less M2 markers compared with macrophages in the peri-tumor areas (101, 102). The temporal and spatial heterogeneity of TAM implies its high plasticity that can be utilized for therapeutic purposes by re-polarization strategies.

## The Impact of TAMs in Human Cancers

Like TANs, the clinical impact of TAMs has not been completely elucidated (**Table 4**). Most clinical studies have linked the density and molecular signatures of TAMs with poor clinical outcomes (113–115). A meta-analysis of literatures by Zhang et al. found that the density of TAMs was associated with poor overall survival (OS) in patients with gastric, urogenital and head and neck cancers with some exceptions in patients with colorectal cancer (113). More recently, deconvolution algorithms were developed to deduce frequencies of different immune cells in bulk tumors, which provided another way to examine potential impact of immune microenvironment (6, 116, 117). According to a few algorithms, subpopulations of TAMs (e.g., M1 vs M2) can be distinguished, and M2 falls into the poor-prognosis category among other immune cells (118). However, single-cell RNA-seq data in human patients suggested that M1- and M2-like features may co-vary at a single cell level, and therefore, the separation between the anti- and pro-tumor TAMs is indistinctive (119). Furthermore, some generic macrophage signatures are highly correlative with T cell and B cell signatures, which are in turn associated with good prognosis (120). Thus, similar to situation of TANs, simple analysis to characterize clinical impact of TAMs as an entirety is confounded by the heterogeneity, plasticity and context-dependency of TAM functions. The simple M1-M2 bi-polarization model, which is derived *in vitro*, is insufficient to fully recapitulate these characteristics *in vivo* (121). Instead, more granular classification and functional characterization may be required before the exact clinical impact of TAMs can be determined in specific clinical contexts.

**Table 4:** The clinical relevance of TAMs in human cancers.

Type of Cancer	Marker	Correlation	Reference
Breast cancer	CD68^+^, CD11c^+^, or CD163^+^	CD163^+^ correlated with reduced OS and DFS; CD11c^+^ in stroma correlated with higher OS and DFS	(103)
Invasive breast cancer	CD68^+^	High tumor grade, negative estrogen receptor	(104)
Bladder cancer	CD68^+^	Invasive subtype, reduced 5-year survival	(105)
Hodgkin’s lymphoma	CD68^+^	Shortened patient survival	(106)
Hepatocellular Carcinoma	CSF-1R	Increased intrahepatic metastasis, tumor recurrence, reduced patient survival	(107)
Advanced thyroid cancer	CD68^+^	Advanced histological grade, tumor invasiveness and mortality	(108)
Non-small cell lung cancer	CD68^+^ in tumor islet and stroma	Increased survival	(109)
Follicular lymphoma	CD68^+^	Reduced OS	(110)
Colon cancer stage II	CD68^+^ and CD206^+^	CD206/CD68 ratio associated with poor DFS and OS	(111)
Head and neck squamous cell carcinoma	Meta-analysis of TAMs and M2 macrophages	Both correlated with poor clinicopathologic markers	(112)

## Tumor-Associated Macrophages in Tumor Initiation

It has been well noted that inflammatory conditions are positively correlated with carcinogenesis (95, 122). Since macrophages are one of the major participants in regulating the inflammation network, its role in tumor initiation has been widely reported. Cytokines IL-23 and IL-17 derived from CD11b^+^ F4/80^+^ are responsible for colorectal cancer initiation and growth (123). Selective ablation of IL-6 in monocytes and Kupffer cells resulted in inhibition of STAT3 signaling and delayed the tumorigenesis in a Mdr2-defecient spontaneous hepatocellular carcinoma model (68). Depletion of Stat3 in CSF1R expressing cells in mice resulted in drastic inflammatory response of the intestine and malignant tumor formation (69). These studies indicate that TAMs play an essential role in tumor initiation.

## Tumor-Associated Macrophages in Angiogenesis

Angiogenesis is crucial to maintain the fast growth of a tumor, especially after it reaches a certain size. Among many supporting factors contributing to angiogenesis in tumors, macrophages play an indispensable role. TAMs produce epidermal growth factor (EGF), fibroblast growth factor (FGF) (124), VEGF (125), transforming growth factor-α and -β (126, 127), Il-1β (128), IL-6, IL-8 (129), platelet-activating factor (130), platelet-derived growth factor (PDGF), thrombospondin-1 (131), MMPs, and other molecules that promote and stabilize the intratumoral blood vessels formation (114). The number of infiltrated macrophages correlates with the vessel density in invasive breast carcinoma (70). Overexpression of CSF-1 and its receptor correlates with poor prognosis in human breast carcinoma (132). CSF-1 was also found to direct macrophage recruitment before malignant initiation and produce VEGF to promote angiogenesis (133). Ablation of VEGFA in myeloid cells could inhibit the angiogenic switch (71). Macrophages can be recruited to hypoxic region of tumor by CCL-2, where upregulated HIF1α/HIF2α orchestrates the transcription of many angiogenesis related genes including VEGF, CXCR4, CCL2, and endothelins which reciprocally enhanced the recruitment of macrophage (72, 134). Genetic deletion of REDD1 under hypoxia can enhance glycolysis in TAMs, which raises the competition of glucose between TAMs and endothelial cells. This prevents the formation of an abnormal vascular network and reduces metastasis (73). Besides producing VEGF, macrophage can also free VEGF by degrading extracellular compartments through MMP9 expressed. Targeting MMP9 of tumor infiltrating macrophages by a bisphosphonate, zoledronic acid, inhibited the angiogenesis in a cervical carcinoma model (74). Tie2^+^ macrophage is one well-characterized subset in primary tumor stroma that regulates the angiogenic switch (135). Forget et al. showed that CSF-1 could increase the Tie2^+^ expressing macrophages and angiogenesis in PyMT mammary tumor bearing mice. They also uncovered that Tie2^+^ expressing macrophages could also augment chemotactic response to endothelial cells expressed angiopoietin-2 (75).

## TAMs in Metastasis

Tumor associated macrophages can direct tumor migration and invasion through regulating genes related to metastasis. CD11b^+^/Gr1^mid/low^ tumor infiltrating monocytes/macrophages can induce the expression of S100A8 and S100A9 in MC38 and Lewis lung carcinoma cells. Ablation of their expression significantly diminished the migration and invasion *in vitro* culture as well as reduced liver metastasis and invasion to adjacent tissues without affecting the subcutaneous tumor growth (76). Sometimes the regulation is not uni-direction but rather a paracrine loop. CSF-1 synthesized by tumor cells and EGF derived from macrophages paracrine loop in MMTV-PyMT model were reported by Wyckoff et al. to cause tumor cells to migrate into surrounding connective tissue. The migration effect of both cell types was abrogated by blocking either CSF-1 or EGF signaling (77). CD163^+^ TAMs derived IL-6 regulated EMT to enhance CRC cells migration and invasion. IL-6 activated JAK2/STAT3 pathway to upregulate FoxQ1 expression, which in turn increased the production of CCL2 to promote macrophage recruitment. This reciprocal loop can be blocked by inhibition of CCL2 or IL6 with reduced macrophage migration and metastasis of CTC (78). TAMs in breast patient samples were activated to an M2-like phenotype. DeNardo et al. reported that CD4^+^ T lymphocytes skew the phenotype and effector function of CD11b^+^ Gr1^-^ F4/80^+^ tumor associated macrophages to promote the invasion and metastasis in MMTV-PyMT mammary carcinoma model by stimulating the EGF signaling (79). The TAMs secreted CCL18 to promote mesenchymal breast cancer cells invasion and migration through their receptor PITPNM3 mediated extracellular matrix adherence (80). Another way TAMs promote tumor migration and invasion is through secreting exosomes, which promotes metastasis related signaling (81). Lan et al. showed that miR-21-5p and miR-155-5p encapsulated in the exosomes derived from M2 macrophages downregulate the expression of BRG1 by binding to its coding sequence to enhance the migration invasion and lung metastasis of colorectal cancer (82).

Although the underlying mechanisms of intravasation are still poorly understood, TAMs were reported to participate in this key step of metastasis. Direct contact enabled macrophages to induce invadopodium formation of breast cancer cells through activating RhoA signaling. This invadopodium facilitated transendothelial migration of MDA-MB-231 cells and patient derived triple negative breast cancer cells TN1 *in vitro* (83). Using intravital real-time imaging, macrophage-mediated vascular permeability and the dissemination of tumor cells into the blood stream was visualized *in vivo*. This permeability and intravasation, was transient and localized where macrophages were present, and was regulated by VEGFA signaling from Tie2^+^ macrophages (84).

Having escaped from the primary site, disseminated tumor cells must survive harsh conditions when infiltrating to and colonizing distant organs. Macrophages are a vital player in preparing the metastasis soils, aiding extravasation, maintaining survival, and stimulating growth of the disseminated tumor cells (86, 136, 137). Kaplan et al. discovered that VEGFR1 expressing bone marrow-derived hematopoietic progenitor cells home to pre-metastatic sites before arrival of disseminated tumor cells through the interaction of VLA-4 and its ligand fibronectin in the resident fibroblasts. Blockade of VEGFR1 or depletion of VEGFR1+ cells from bone marrow could abrogate the formation of pre-metastatic niche and prevent metastasis of Lewis Lung carcinoma (136). CYP4A^+^ TAMs infiltration was positively correlated with formation and metastasis. Inhibition of CYP4A showed decreased VEGFR1^+^ myeloid cell recruitment and pro-metastatic protein expression in lung pre-metastatic niche, accompanied by skewing from M2 to M1 polarization in the 4T1 spontaneous metastasis breast cancer model and the B16F10 melanoma model (138). Deletion of S1P receptor 1 (S1pr1) in CD11b^+^ CD206^+^ TAMs reduced the NLRP3 expression and IL-1b production, and thus prevented pulmonary metastasis and tumor lymphangiogenesis in breast tumors (139). Qian et al. showed that tumor cells in contact with macrophages had a higher rate of extravasation. Depletion of macrophages using L-clodronate significantly reduce the extravasation of tumor cells (85). Gr1+ monocyte-derived VEGF promoted the extravasation of breast tumor cells. These monocytes also recruited to pulmonary metastases driven by CCL2 to promote the seeding of PyMT breast cancer cells (86). Kitamura et al. found that CCL2-CCR2 signaling promoted the secretion of CCL3 from metastasis associated macrophages (MAM), which increased the retention of MAM to promote lung metastasis in breast tumor models (87). Su et al. elucidated that mesenchymal breast cancer cells activated macrophages in the vicinity to skew towards a TAM-like phenotype through GM-CSF. The activated TAMs secreted CCL18 could reciprocally induce cancer cell EMT both *in vitro* and *in vivo*. Blockade of either GM-CSF or CCL18 can break this positive feedback loop, and thus reduced metastasis (88). Another study featuring the antitumor effect of TAMs revealed in a mouse model of spontaneous melanoma expressing human RET oncogene that reactive oxygen species was an essential mechanism underlying the tumor proliferation inhibition of CD11b^+^ Ly6C^+^ monocytes. Regulatory CD4^+^ T cell derived IL-10 facilitated tumor progression through inhibiting the recruitment or differentiation of inflammatory monocytes in skin (89). Taken together, numerous lines of evidence support the pivotal roles of TAMs in metastasis, and the underlying molecular mechanisms appear to be diverse and complicated. Therefore, it will be crucial to identify targetable molecules that are key in each specific biological context.

## Clinical Relevance of TAMs and TANs

Multiple strategies are being pursued to target TAMs. One category of clinical trials is to target the CCR2-CCL2 axis, the major chemokine axis responsible for monocyte recruitment. Several clinical trials targeted CCL2 transiently (NCT00992186, NCT01204996, NCT00537368) with Carlumab, and showed acceptable tolerance and preliminary antitumor response in some solid tumors. In combination with chemotherapeutic agent Folfirinox, a CCR2 inhibitor PF-04136309 exhibited benefit in patients with pancreatic cancer (NCT01413022). Depletion of macrophages is another strategy used by many clinical trials. The colony stimulation factor CSF1R signaling is important in regulating macrophage proliferation and survival as well as macrophage recruitment and polarization. Various CSF1R inhibitors were developed and used alone or in combination with other agents for different type of cancers (140). The caveat of macrophage depletion is toxicity, especially for liver cells (141), which highlights the need for more precise targeting of TAMs instead of normal macrophages. Since many studies showed that TAMs resemble the alternative activated M2 phenotypes that favor tumor progression, another strategy is to reprogram M2 to pro-inflammatory M1 macrophage. For instance, CD40 monoclonal antibody was reported to increase the pro-inflammatory factors (M1-promoting) and regulate innate and adaptive immune response (142). As a human immunoglobulin (IgG2) anti-CD40 monoclonal antibody, CP-870893 can specifically target the non-ligand binding site of CD40 and enhance the secretion of IL-12, IL-23, and IL-8. In combination with gemcitabine, CD40 was associated with antitumor activity in PDA patients (143). SEA-CD40 is an agonistic non-fucosylated humanized IgG1 CD40 antibody with enhanced FcγRIIIa binding. It showed superior effect over other CD40 antibodies. The phase I clinical trial in patients with relapsed or refractory metastatic solid tumors are ongoing (NCT02376699). Inhibition of PI3Kγ has been shown to induce proinflammatory gene expression in TAMs without affecting their accumulation in tumors. Suppression of tumors has been shown in some preclinical studies (144). In combination with nivolumab, the PI3Kγ inhibitor is undergoing Phase 1b clinical trial for solid tumors (NCT02637531) with the repolarization of macrophages will be assessed. Ibrutinib, with its inhibition on BTK downstream of PI3Kγ, can induce proinflammatory polarization of macrophages as well as CD8^+^ T cells infiltration. It is in clinical trials in combination with several chemotherapeutic agents to treat pancreatic adenocarcinoma relapsed or refractory solid tumors (NCT02599324, NCT02436668, NCT02303271). Because TLRs polarize macrophages towards more proinflammatory phenotype, their agonists can be used to induce immune response against tumors. Several TLR agonists (TLR4, 7/8, 9) are in clinical trials in combination with different immune checkpoint blockade (140). Another unneglectable strategy is to unleash the phagocytosis of macrophages that was compromised in tumors. CD47 is a receptor for thrombospondin on human myeloid and endothelial cells. It protects the host cells from destruction by macrophages through binding to SIRP1a on macrophages. Targeting CD47 by antibody or other agents can stimulate phagocytosis of tumor cells in many mice models. Hu5F9-G4, a human monoclonal antibody that targets CD47 is under clinical trial against solid tumors (NCT02216409, NCT02953782). Another new agent TTI-621, a SIRPa-Fc fusion protein, is being tested for solid tumors in Phase I clinical trials (NCT02890368).

Despite the increasing recognition of importance of TANs, clinical trials that specifically focus on neutrophils are only in their fetal stage. Several drugs currently tested may have potential impact on TANs. For instance, some neutrophil elastase inhibitors, PDE5 inhibitors and COX2 inhibitors, were reported to inhibit the pro-tumor activity of neutrophils (NCT01170845, NCT02544880, NCT00752115). In addition, TGF-β was reported to skew neutrophils to a more protumor phenotype (20), and TGF-β inhibitors may stimulate neutrophil to antitumor phenotype (13). Other drugs are being tested to reduce TAN recruitment or induce TAN apoptosis. Several chemotaxis inhibitors, such as those targeting CXCR2 and CCR5, are under investigation to hinder the recruitment of neutrophils to the TME (NCT02370238, NCT02001974, NCT03274804, NCT01736813). Trail receptor expressed by neutrophils can be agonized to induce their apoptosis (NCT01088347, NCT00508625, NCT00092924). CD47-SIRPα inhibitors and CD40 monoclonal antibody that regulate TAMs could also limit the migration of neutrophils to tumor or deplete neutrophils (NCT02216409, NCT03717103, NCT02367196, NCT01103635). The clinical outcome of these above agents will provide invaluable insights into the roles of TANs in human tumors.

## The Relationship Between TANs, TAMs, and Myeloid-Derived Suppressor Cells (MDSCs)

By definition, MDSCs are immunosuppressive and can blunt T cell cytotoxicity to create a favorable microenvironment for tumor growth. Blocking the immunosuppression of MDSCs will benefit antitumor response and improve the efficacy of the immunotherapies. Two different subgroups of MDSCs were identified in both mice and human: polymorphonuclear MDSCs (PMN-MDSCs) and monocyte MDSCs (M-MDSCs). The PMN-MDSCs resemble neutrophils in morphology and phenotypes and are defined as CD11b^+^ Ly6G^+^ Ly6C^low^ in mice and CD11b^+^ or CD3^+^, CD15^+^ or CD66b^+^, and CD14^-^ in human. The M-MDSCs resemble monocytes and are identified as CD11b^+^Ly6G^-^Ly6C^high^ in mice and CD11b^+^ or CD33^+^, CD14^+^, and HLA-DR^low^ in human (145). They use different mechanisms for immunosuppression with M-MDSCs more potent than PMN-MDSCs per cell but PMN-MDSCs typically outnumbering M-MDSCs. The major immunosuppressive molecules involved in their activities are ARG1, NO, ROS, prostaglandin E2, which are similar to those used by M2 macrophages or N2 neutrophils to promote tumor progression (145, 146). Thus, the major question is if and how MDSCs differ from TANs and TAMs.

While TAMs and TANs usually refer to macrophages and neutrophils infiltrating tumors, MDSCs are systemically accumulated in tumor-bearing hosts. They are derived from the bone marrow under the remote influence of tumors, and can be found in peripheral blood and spleen, in addition to the tumor microenvironment.

PMN-MDSCs are recognized using the same set of markers for neutrophils both in mice and human, although in some circumstances PMN-MDSCs can express unique markers distinct from normal neutrophils (147). As TANs are a heterogenous population that may have anti-tumor or pro-tumor functions, PMN-MDSCs are more likely the pro-tumor subset of TANs (145). It is worth noting that PMN-MDSCs and neutrophils can be distinguished in human peripheral blood since the former are enriched in low density Ficoll gradient fraction while the latter are in the high density fraction (145, 148).

The markers used to identify M-MDSCs in mice are different with TAMs in that M-MDSCs has high expression of Ly6C while TAMs are recognized as high expression of F4/80, intermedium to low expression of Ly6C, and undetectable expression of S100A9. Unlike normal monocytes, M-MDSCs do not express or have low expression of HLA-DR (149).

MDSCs also exhibited considerable plasticity in TME. M-MDSCs had the potential to differentiate into PMN-MDSCs as reported by Youn et al, where the pathway for monocyte differentiation was dysregulated to preferentially generate G-MDSCs (150). MDSCs can also generate M2 TAMs and N2 TANs. Kuma et al. reported that STAT3 regulated the differentiation of MDSCs into immunosuppressive TAMs in hypoxic conditions (151). TGF-β secreted by MDSCs and other tumor stromal cells can deviate neutrophils into N2 TANs, which in turn recruit Treg cells through CCL17 secretion (20). The plasticity of MDSCs is also reflected by their ability to trans-differentiate into myeloid cells in different lineages. In a study by Corzo et al, MDSCs from spleen could differentiate into both macrophages and dendritic cells (DCs) while MDSCs from tumor only differentiated into macrophages. MDSCs from these two sites also differed in their T cells suppression ability. The spleen MDSCs suppressed only CD8^+^ T cells while the tumor MDSCs suppress both antigen specific and antigen non-specific T cells (152). Thus, increased plasticity and potency for differentiation may be a general feature of MDSCs as compared to TAMs and TANs.

It is still premature to draw a concrete conclusion on the relationship between MDSCs and TANs and TAMs. However, profiling these cells at genomic and proteomic levels will facilitate solving the myth of MDSCs (153–155). Clear description of the context and markers used to study these populations is the best practice for current researches in the field of oncoimmunology (148, 156).

## Interactions Between TANs, TAMs, and Tumor-Infiltrating Lymphocytes

TAMs and TANs extensively interact with tumor infiltrating lymphocytes, and have pleotropic effects. Several examples are provided below with a common theme that both TAMs and TANs use multiple overlapping pathways to crosstalk with T cells, including engagement of immune checkpoints and secretion of cytokines.

ROS and arginase I released by TANs inhibited T cell activation and proliferation (7, 20, 157). Arginase I produced by TANs blunt T cell response in human renal cancer carcinoma and non-small cell lung cancer (158, 159). TANs also induced apoptosis of non-activated CD8^+^ T cells through NO and TNF-α (160). Immune checkpoints can be activated on T cells by their ligands expressed on TANs. High level of PD-L1 was expressed on TANs in patients with gastric cancer induced by tumor secreted G-CSF. These activated PD-L1^+^ neutrophils suppressed the T cells function *in vitro* and is correlated with disease progression and patient mortality (161). PD-L1^+^ neutrophils were also found in peritumor site of patient samples of hepatocellular carcinoma and was associated with poor disease free patient survival (162). Other than the immunosuppressive effect, Ponzetta et al. reported that neutrophils drove the polarization of a subset of unconventional CD4^-^ CD8^-^ αβ T cells in a IFN-γ dependent way to resist 3-methycholantrene induced murine sarcomas in mice (163).

TAMs exert immunosuppressive effect through several mechanisms (8). Arginase I and iNOS expression by TAMs partially regulated their T cell suppressive activity (164). Genetic depletion or pharmaceutical inhibition of TAMs and CSF-1 restored the cytotoxic CD8^+^ T cell functions with tumor regression in mouse mammary and cervical models (165). Similar to TANs, T cell immune checkpoint ligands were also found to be expressed on TAMs. Circulating monocytes and TAMs in patients with glioblastoma expressed increased level of B7-H1. *Ex vivo* stimulation monocytes with conditional medium resulted in increased production of IL-10 which upregulated B7-H1 expression. These stimulated monocytes induced T cells apoptosis in co-culture (166). Tumor associated macrophages were found to be a primary source for PD-L1 in mouse and human cholangiocarcinoma, where inhibition TAMs and G-MDSCs improved immune checkpoint blockade efficacy (167). Regulatory T cells were also used by TAMs to suppress T cell immunity. Natural regulatory cells (nTreg) were recruited by TAMs to suppress the effector function of CD4^+^ and CD8^+^ T cells (168, 169).

## Interactions Between TAMs and TANs

Arising from a common progenitor lineage, the multifaceted roles of TANs and TAMs are implicated in almost every steps of tumor growth and metastasis. However, there are still few studies on the interactions between TANs and TAMs in the tumor microenvironment settings (170, 171). Recently, emerging studies began to integrate both populations to gain a better understanding of their interactions in the varying tumor microenvironment. Kumar et al. demonstrated in a series of mouse tumor models a significant increase of infiltrated PMN-MDSCs (CD11b^+^ Ly6C^1o^ Ly6G^+^) in their attempt to deplete TAMs by pharmaco-inhibition or antibody neutralization of CSF1R. The infiltrated PMN-MDSCs recruited by carcinoma associated fibroblasts failed the expected therapeutic effect of CSF1R inhibition (172). Concomitantly, Janiszewska et al. found that minor subclones of breast tumor cooperated to drive breast tumor metastasis through inducing local and systematic stimulation of pro-metastatic neutrophils (CD11b^+^ Ly6C^1o^ Ly6G^+^). Neutrophils were significantly higher in blood, primary tumors and lungs induced by an IL11-expressing minor subclone of MDA-MB-468. Although the percentage of macrophages in primary tumors was not shown, it did decrease in blood and lungs (173). Using a panel of eight mouse triple negative breast cancer models, our recently published paper revealed that tumors did not recruit TANs or TAMs equally. Even as the same subtype of breast cancer, they could still be immuno-subtyped into neutrophil-enriched subtype (NES, CD11b^+^ Ly6C^mid^ Ly6G^+^) or macrophage-enriched subtype (MES, CD11b^+^ Ly6G^-^ Ly6C^-^ F4/80^+^) according to their preference to recruit TANs and TAMs. A mutual exclusion between TANs and TAMs was observed. When one was depleted the other would be up-regulated (174). The mechanism underlying this mutual exclusion awaits further investigation. Yet, it shed light on a possible co-evolution between tumor associated myeloid cells and tumors.

## Breast Cancer and Its Microenvironment

Breast cancer is the most common malignancy among women. Breast cancer is heterogeneous with distinct molecular and histological features that can be ascribed into luminal A-like (ER^+^, PR^+^, HER2^-^), luminal B-like HER2^-^, luminal B-like HER2^+^, HER2-enriched (non-luminal) and triple-negative (ER-, PR-, HER2-) in current clinical practice (175). The heterogeneity of the subtypes influences treatment decision as well as the therapeutic outcomes. For instance, six detailed subclasses with distinct sensitivity to therapeutic drugs have been characterized (176). The heterogeneity exists not only across full spectrum of breast tumors as inter-tumoral but also between different regions of the tumor. Plus, molecular signatures evolve along the pressure from the microenvironment during progression as well as from the therapeutic intervention (177, 178).

Stromal cells, on the other hand, also bare heterogeneity between different tumors or within the same tumor. Tumor intrinsic signaling is one of the major factors that determines the heterogeneity of microenvironment. Studies showed that inflammatory response is downstream intrinsic oncogenic pathways (179, 180). And local production of chemokines and cytokines from cancer cells regulate the tumor infiltrating immune constituents of the microenvironment. A dichotomy of immune microenvironment was reported in different lung cancer subtypes. Macrophages are predominantly present in Kras adenocarcinoma models while neutrophils were mainly recruited to the squamous cell carcinoma region by Lkb1 and Pten inactivation but not the adjacent adenocarcinoma region (181). Our group discovered that mTOR signaling in cancer cells determines the MDSC accumulation through regulating G-CSF production. This MDSC accumulation preferentially occur in tumor models exhibiting elevated mTOR activities (182). More recently, we further demonstrated a dichotomous myeloid cell profiles across eight murine triple negative breast cancers; some of the tumors are enriched with TAMs with few TANs and some others are enriched with TANs with a minority of TAMs. We named these tumors as macrophage-enriched and neutrophil-enriched subtypes (MES and NES) respectively. This dichotomy may be driven by two forces: 1) the intrinsic properties of cancer cells, such as the mTOR activities and EMT (changing the EMT status of the tumor cells could alter the type of myeloid being recruited as shown in our work), and 2) the mutually negative impact between TAMs and TANs. Interestingly, when MES tumors that are initially sensitive to therapies acquire resistance, a shift toward NES was observed, indicating the plasticity of myeloid compartment during therapeutic interventions (174).

## Concluding Remarks and Open Questions

**Figure 1:**
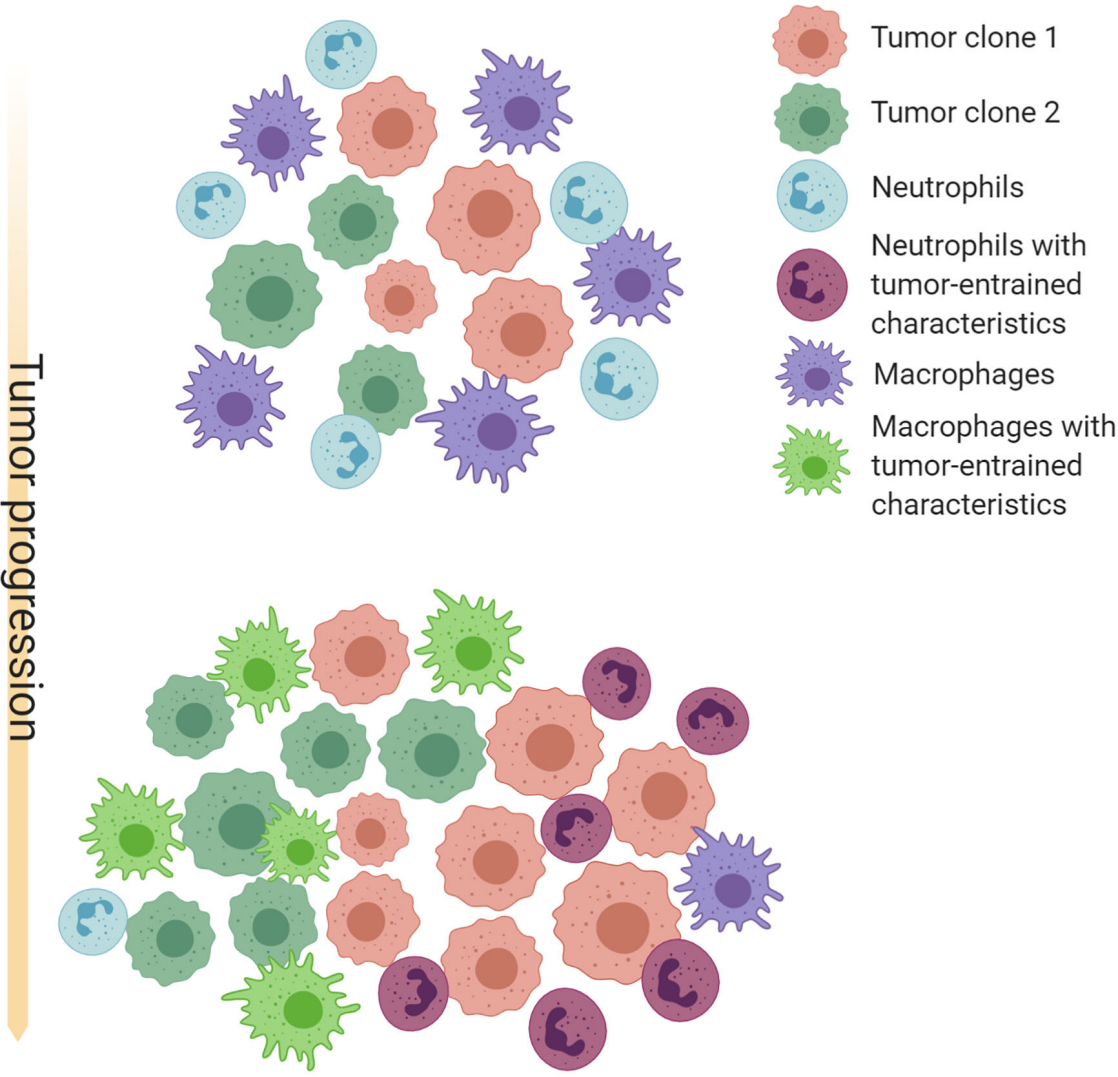
Schematic illustration of possible co-evolution among tumor cells, TANs and TAMs. Cancer cells with different genetic or epigenetic traits may selectively recruit neutrophils or macrophages, and provide an initial milieu to influence differentiation, polarization, and survival of these myeloid cells. TAMs and TANs in turn confer selective benefit to some clones by paracrine and direct cell-cell interactions. TAMs and TANs may also compensate each other and compete for the same microenvironment niches. These interactions may often result in a feed-forward loop that favor an equilibrium of TAM-enriched or TAN-enriched microenvironment, as well as specific cancer cell-intrinsic characteristics. Thus, co-evolution with TAMs and TANs may be an important force driving intra- and inter-tumor heterogeneity. Created with Biorender.com.

The progression of tumorigenesis and metastasis resembles the evolution of ecosystems. On one hand, tumor cells are under constant selective pressure skewing towards increased survival and proliferation (183, 184). On the other hand, tumors continuously reprogram TME systematic environment to create an abnormal ecosystem. Extensive molecular evolution of tumor-associated stroma during cancer progression has been shown by gene expression analysis (185, 186). A possible co-evolution pattern of TANs, TAMs and tumors is shown in **Figure 1**.

TANs and TAMs participate in many steps of tumor progression and metastasis. As a major part of the innate immune system, they have drawn tremendous interest to their roles in almost every step of tumor progression and metastasis. Despite this knowledge, several questions remain outstanding.

First, do the frequencies and functional roles of TAMs and TANs vary across individual tumors? As discussed in previous sections, both TAMs and TANs can play opposite roles in different contexts. Variable polarization status may create a continuous spectrum between anti- and pro-tumor functionalities. The exact positioning of TAMs and TANs in this spectrum will likely be influenced by cancer cells and other immune cells. In our previous studies, mTOR and EMT pathways were found to contribute to enrichment of TANs and TAMs in different models, respectively. Moreover, depletion of TANs led to increase of monocyte infiltration whereas depletion of TAMs resulted in influx of TANs. Thus, the frequencies of these myeloid cells in a particular tumor are jointly determined by tumor-intrinsic factors and their mutual (negative) impacts. In terms of functions, genetic depletion of macrophages from different MES models had opposite or highly distinct effects on tumor growth and therapeutic responses to checkpoint blockade therapies. Thus, additional factors seem to dictate TAM polarization independent of recruitment. In general, models or biological contexts have not been sufficiently considered as an important variable in understanding the roles of TAMs and TANs, which severely prevent the integration of our knowledge.

Second, how does intertumoral TAM and TAN variations correlate with known subtypes of tumors? A general classification of “hot” versus “cold” tumors has been used to describe tumors with or without immune cell infiltration (especially T cells). However, not all hot tumors are similar – the exact composition of the immune TME including TAMs and TANs should be considered independent of lymphocytes, as they may use totally distinct immunosuppressive mechanisms. Triple negative breast cancers have been shown to be heterogeneous and can be further divided into 4–6 different subtypes based on cancer-intrinsic gene expression (176, 187). However, the characteristics of some subtypes are clearly related to activation and suppression of immune system. Furthermore, the correlation between EMT and macrophages has been uncovered in a number of studies (88, 174, 188, 189), indicating a link between metaplastic histology or “claudin-low” phenotype (190) and macrophage-enriched TME. Taken together, these lines of evidence support correlations between tumor-intrinsic heterogeneity and TME heterogeneity.

Finally, how do tumor cells and immune cells co-evolve as an integrated ecosystem? The concept of immunoediting has greatly facilitated our understanding of interactions between tumor cells and the immune system (191). The selective pressure exerted by anti-tumor immunity impacts clonal evolution and ultimately leads to escape of immunosurveillance. Moreover, cancer cells also gain additional selective advantages by turning immune cells into conspirators in tumor progression (137). Thus, the crosstalk between cancer and immune cells is bidirectional, forming the foundation of co-evolution. It should be noted that this co-evolution may take a distinctive path in each individual tumor, resulting in a unique ecosystem. TAM and TAN may together provide examples illustrating this process. For instance, mesenchymal-like tumor cells are more likely to recruit TAMs, which in turn reinforce mesenchymal properties. Both mesenchymal stem cells and TAMs may repel or compete against infiltration of TANs, thereby forming a macrophage-enriched TME (174). The mTOR pathway, on the other hand, stimulates systemic and local accumulation of neutrophils, which might outcompete macrophages and drive tumor evolution toward another direction (174, 182). More in-depth and mechanistic studies are required to test these hypotheses. Furthermore, the clinical implications also need to be explored to facilitate better immunotherapies.

In conclusion, the precise influence of TAMs, TANs and other immune cells on tumor progression and metastasis needs to be collectively analyzed together with tumor-intrinsic properties to reveal molecular mechanisms underlying the coevolution in context-dependent manners.

## Author Contributions

LW and XZ conceived and wrote the manuscript. All authors contributed to the article and approved the submitted version.

## Funding

XZ is supported by Breast Cancer Research Foundation and McNair Medical Research Institute.

## Conflict of Interest

The authors declare that the research was conducted in the absence of any commercial or financial relationships that could be construed as a potential conflict of interest.
